# Evaluación clínica e imagenológica de la articulación temporomandibular en pacientes sometidos a condilectomía como tratamiento de hiperplasia unilateral. Estudio de serie de casos

**DOI:** 10.21142/2523-2754-0904-2021-090

**Published:** 2021-12-09

**Authors:** Héctor Andrés Laguna-Monagas, Adalsa Hernández-Andara, Ana Ortega-Pertuz, Hans Cordsen, Ronar Gudiño, Carlos Contreras

**Affiliations:** 1 Programa de Cirugía y Traumatología Bucal y Maxilofacial, Hospital General del Oeste Doctor José Gregorio Hernández. Caracas, Venezuela. hectorlaguna92@gmail.com Programa de Cirugía y Traumatología Bucal y Maxilofacial Hospital General del Oeste Doctor José Gregorio Hernández Caracas Venezuela hectorlaguna92@gmail.com; 2 Unidad de Diagnóstico por Imagen, Clínica Félix Boada. Caracas, Venezuela. adalsa1@yahoo.com Unidad de Diagnóstico por Imagen Clínica Félix Boada Caracas Venezuela adalsa1@yahoo.com; 3 Instituto de Investigaciones, Facultad de Odontología de la Universidad del Zulia. Maracaibo, Venezuela. anitaortegav@gmail.com Universidad del Zulia Instituto de Investigaciones Facultad de Odontología de la Universidad del Zulia Maracaibo Venezuela anitaortegav@gmail.com; 4 Servicio de Cirugía Maxilofacial, Hospital General del Oeste Doctor José Gregorio Hernández. Caracas, Venezuela. hanswcmaxilofacial@hotmail.com, odronar@gmail.com, carlosalbertocontrerasdelgado@gmail.com Servicio de Cirugía Maxilofacial Hospital General del Oeste Doctor José Gregorio Hernández Caracas Venezuela hanswcmaxilofacial@hotmail.com odronar@gmail.com carlosalbertocontrerasdelgado@gmail.com

**Keywords:** cóndilo mandibular, articulación temporomandibular, hiperplasia, tomografía computarizada, mandibular condyle, temporomandibular joint, hyperplasia, computed tomography

## Abstract

La hiperplasia condilar unilateral (HCU) es un crecimiento excesivo, no neoplásico y autolimitante del cóndilo mandibular que se suele iniciar durante la pubertad, con predilección en las mujeres, y se considera una aberración del mecanismo de crecimiento normal del cóndilo. Este crecimiento anormal continúa hasta mediados de los 20 años y produce prognatismo mandibular, asimetría facial y oclusal, con el desplazamiento progresivo de la mandíbula al lado contralateral. El propósito de este reporte fue describir dos casos de HCU tratados con condilectomía alta (CA) y cirugía ortognática en pacientes del sexo femenino (23 y 25 años), con énfasis en sus aspectos clínicos e imagenológicos y su seguimiento posquirúrgico tardío. Ambas pacientes presentaron resultados estéticos satisfactorios, sin dolor/ruido relacionado con la articulación temporomandibular, apertura bucal dentro del rango de normalidad y relación canina y molar clase I. En la tomografía computarizada, se observaron signos de remodelación en el cóndilo intervenido. La CA combinada con cirugía ortognática es un tratamiento adecuado en casos de hiperplasia unilateral, lo que devuelve la funcionalidad y la estética al paciente. La remodelación ósea observada en los cóndilos intervenidos parece indicar que la cabeza mandibular mantiene su capacidad adaptativa aún en pacientes adultos.

## Abstract (en)

Unilateral condylar hyperplasia is a non-neoplastic and self-limiting overgrowth of the mandibular condyle that usually begins during puberty, predominates in women and is considered an aberration of the normal growth mechanism of the condyle. This abnormal growth continues until the mid-20s and produces mandibular prognostism, facial and occlusal asymmetry with progressive displacement of the mandible to the contralateral side. The purpose of this report was to describe the cases of two female patients (23 and 25 years old) with unilateral condylar hyperplasia treated with high condylectomy and orthognathic surgery, with emphasis on clinical and imaging aspects and late post-surgical follow-up. Both patients presented satisfactory cosmetic results, without pain / noise related to the temporomandibular joint, mouth opening within the normal range, and class I canine and molar relationship. Computed tomography showed signs of remodeling in the affected condyle. High condylectomy combined with orthognathic surgery is an adequate treatment in cases of unilateral hyperplasia, restoring functionality and aesthetics to the patient. The bone remodeling observed in the intervened condyles seems to indicate that the condylar head maintains its adaptive capacity even in adult patients.

## INTRODUCCIÓN

El término hiperplasia se refiere al aumento de la producción y el crecimiento de células normales en un tejido u órgano sin un aumento del tamaño celular, conservado su forma básica ^1^. La hiperplasia condilar (HC) describe, en general, todas las condiciones que causan crecimiento excesivo y progresivo del cóndilo mandibular ^1^^,^^2^. 

La HC fue clasificada por Obwegeser y Makek ^3^, de acuerdo con el vector de crecimiento, en tres subtipos: vertical, horizontal o combinada. En el tipo I se produce una elongación hemimandibular, el tipo II se refiere a una hiperplasia hemimandibular y el tipo III es una combinación de ambas. Wolford *et al*. ^1^ proponen una clasificación que incluye las diversas patologías que causan una HC. El tipo 1 suele presentarse durante la pubertad, es autolimitante y se considera una aberración del mecanismo de crecimiento condilar normal, que causa un vector de crecimiento de predominio horizontal que resulta en prognatismo mandibular que puede ser bilateral (tipo 1A) o unilateral (tipo 1B); dicho crecimiento termina entre principios y mediados de los 20 años.

El tipo 2 se refiere al agrandamiento del cóndilo causado por un osteocondroma y puede desarrollarse a cualquier edad. Produce un crecimiento excesivo vertical unilateral de la mandíbula y la cara; el proceso de crecimiento puede continuar indefinidamente, con un empeoramiento progresivo de la asimetría facial. En el tipo 3 se clasifican otros tipos de tumores benignos que pueden causar agrandamiento condilar, como osteoma, neurofibroma, tumor de células gigantes, displasia fibrosa, condroma, condroblastoma y malformación arteriovenosa. En el tipo 4 se encuentran los tumores malignos de crecimiento en el cóndilo mandibular (condrosarcoma, mieloma múltiple, osteosarcoma, sarcoma de Ewing y lesión metastásica) ^1^. 

La HC unilateral (HCU) ha mostrado predilección por el sexo femenino (razón 2:1), lo que sugiere una influencia estrogénica ^4^^-^^6^. Su etiología ha sido relacionada con factores genéticos, ambientales, funcionales, hormonales ^5^, endocrinos ^7^, artrosis ^2^ o infección articular ^8^. En general, las causas son poco conocidas, pero se describe la multiplicación anormal de los precondroblastos en la zona proliferativa del cartílago condilar; la capa cartilaginosa entonces sería más gruesa y el número de islotes cartilaginosos mayor de lo habitual ^9^. 

Clínicamente, los pacientes con HCU presentan un crecimiento mandibular acelerado que continúa hasta mediados de los 20 años, asimetría facial y oclusal, con un desplazamiento progresivo de la mandíbula hacia el lado contralateral, mordida cruzada posterior unilateral en el lado contralateral ^1^ o sobreerupción de los dientes superiores en busca de lograr la oclusión ^5^. Asimismo, se observa inclinación transversal del cuerpo mandibular en el lado afectado, aplanamiento transversal del cuerpo mandibular en el lado contralateral y empeoramiento de la oclusión unilateral clase III en el lado ipsilateral ^1^. Por otro lado, existe una compensación en el tercio medio facial por alargamiento del proceso alveolar y escaso desvío del mentón ^5^. Los ángulos goniales son obtusos, acompañando una forma facial más triangular y menos cónica ^10^. Funcionalmente, la HC puede cursar con dificultades fonéticas, de masticación y deglución relacionada con la desarmonía oclusal, obstrucción nasal por desviación del tabique nasal e hipertrofia de cornetes, trastorno de la articulación temporomandibular, problemas estéticos y de autoestima ^8^.

El diagnóstico de la HCU se realiza mediante la asociación de signos clínicos y radiológicos, puede ser corroborada por exámenes de medicina nuclear y confirmada mediante el estudio histopatológico cuando el paciente es sometido a intervención quirúrgica ^5^^,^^10^. La evaluación imagenológica suele evidenciar cabeza y cuello condilar alargados con una morforfología relativamente normal; en una vista coronal, la superficie articular aparece más redondeada que lo normal. El cuerpo mandibular se alarga, la altura vertical del cuerpo mandibular posterior puede estar disminuida, el grosor anteroposterior de la sínfisis y el alvéolo puede ser más estrecho. La anchura medio-lateral y la dimensión anteroposterior de las ramas, usualmente, son menores que lo normal; la longitud de la base del cráneo tiende a disminuir y la angulación de la base del cráneo (silla turca a nasión y nasión a basión) a aumentar; la pendiente del borde posterior de la rama, en comparación con el plano horizontal de Frankfort, puede tener un ángulo anterior mayor al normal ^1^^,^^10^. 

En particular, la tomografía computarizada (TC) ha ganado interés en el diagnóstico de deformidades craneofaciales debido a la ventaja del examen en los tres planos del espacio ^4^^,^^5^^,^^9^^,^^11^ y a que ofrece el uso de herramientas de *software* que permiten mediciones precisas, lo que es útil para evaluar alteraciones morfológicas ^5^^,^^9^. En el caso de la HC, la TC posibilita, además, determinar si el agradamiento condilar es generalizado y proporcional, o localizado desde un cóndilo normal; esto último es característico de un osteocondroma. El análisis volumétrico del caso es valioso en la planificación quirúrgica, con resultados más predecibles ^7^.

El diagnóstico diferencial de HC incluye una relación esquelética de clase III con crecimiento mandibular y maxilar normal, un crecimiento maxilar deficiente con una mandíbula normal, o HC tipo 1 con o sin crecimiento maxilar deficiente ^1^. La HC tipo 1 debe ser diferenciada de otros procesos que causan asimetría facial, como microsomía hemifacial, atrofia hemifacial, anquilosis y tumores óseos, macrognatismo unilateral (hipertrofia mandibular unilateral) y laterognatia. En el diagnóstico clínico diferencial también se consideran neoplásicas, tales como osteocondroma, osteoma, osteblastoma y condrosarcoma, las cuales pueden mostrar características clínicas, radiográficas y oclusales similares a la HCU ^5^^,^^7^.

La modalidad de tratamiento de la HCU es compleja y debe considerar múltiples factores, como la edad del paciente, la gravedad de la asimetría, la maloclusión y la actividad del crecimiento condilar ^1^^,^^2^^,^^5^^,^^12^^,^^13^. Entre las opciones de tratamiento se sugieren la osteoplastia del cóndilo mandibular (2-3 mm), la condilectomía alta (CA) (4-5 mm) o la condilectomía baja (8-12 mm), y es la CA la que permite retirar la superficie cartilaginosa y el hueso subcondral, eliminando el centro de crecimiento ^8^. El tratamiento de elección en una HCU activa incluye CA, reposicionamiento del disco articular y cirugía ortognática ^(1, 2, 10)^. En pacientes adultos con síntomas progresivos de asimetría facial y mordida abierta sin crecimiento condilar activo, se aconseja la cirugía ortognática ^2^.

Imagenológicamente, se han observado cambios posquirúrgicos en los cóndilos intervenidos por HCU que sugieren neoformación ósea, así como presencia de erosión, esclerosis, quiste subcondral y fragmentos óseos por delante de la cabeza condilar ^5^^,^^9^^,^^14^^-^^17^. De acuerdo con lo anteriormente expuesto, este trabajo presenta dos casos de HCU tratados con CA y cirugía ortognática, con énfasis en sus aspectos clínicos e imagenológicos, y su seguimiento posquirúrgico tardío. 

## REPORTE DE CASOS

### Caso 1

Paciente femenina de 25 años de edad, sin antecedentes médicos contribuyentes, quien señala el inicio de enfermedad actual a los 16 años, cuando se evidenció el crecimiento asimétrico de la mandíbula. A los 20 años acudió al ortodoncista, quien diagnosticó deformidad dentoesqueletal (DDE) clase III, motivo por el cual inició un tratamiento con ortodoncia prequirúrgica. Posteriormente, fue referida a interconsulta con el Servicio de Cirugía y Traumatología Bucal y Maxilofacial (SCM) del Hospital General del Oeste Doctor José Gregorio Hernández (Caracas, Venezuela), para evaluación y conducta. 

El examen funcional de la paciente mostró desviación mandibular hacia la izquierda, ruidos articulares en apertura y cierre en ambas articulaciones temporomandi-bulares, dolor de moderada intensidad de acuerdo con la escala visual analógica ^18^ (EVA = 7) a la masticación de alimentos sólidos y en apertura máxima en región preauricular derecha. Durante el examen clínico de la ATM, se evidenciaron en el lado derecho, ruidos crepitantes en apertura temprana y cierre tardío; en el lado izquierdo se verificó clic en apertura y cierre tardío, así como deflexión de 1 mm hacia la derecha en apertura y desviación hacia la izquierda en cierre. La apertura bucal estuvo conservada, la paciente indica dolor en apertura máxima, esta última cuantificada en 45 mm. 

**Figura 1:**
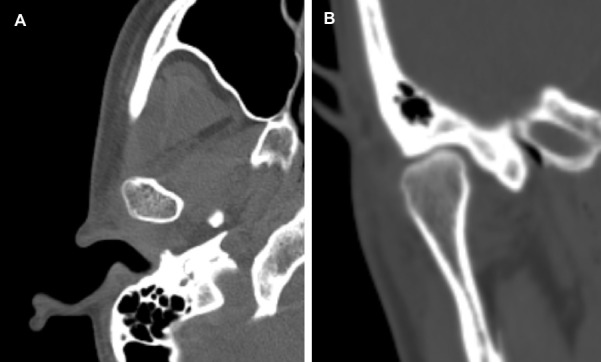
Imágenes prequirúrgicas de tomografía computarizada del caso 1. A. Corte axial de cabeza mandibular derecha, presentado densidad ósea homogénea. B. Corte coronal donde se evidencian características morfológicas y densidad ósea dentro de los patrones imagenológicos de normalidad.

Se observó al examen intrabucal, aparatología ortodóntica en posición maxilo-mandibular con topes oclusales en unidades dentarias 1.7 y 2.7, relación molar clase I bilateral, relación canina clase III derecha y clase II izquierda. Línea media dental maxilar desviada 2 mm hacia la izquierda, línea media dental mandibular desviada 3 mm hacia la derecha, ambas con respecto a línea media facial e inclinación del plano oclusal. Mediante el examen imagenológico con TM, se observó la elongación del cóndilo mandibular derecho (Figura 1). Las características imagenológicas de la ATM del lado izquierdo estaban dentro de los patrones imagenológicos de normalidad.

Se planificó una cirugía ortognática en la que se realizó una osteotomía Le Fort I de impactación posterior derecha de 7 mm e izquierda de 2 mm, mentoplastia de traslación de 5 mm a la derecha y avance de 3 mm, abordaje preauricular para realizar CA de 5 mm y discopexia de disco articular derecho con sutura seda 2-0, el cual se encontraba desplazado anterolateralmente. Se realizó el estudio histopatológico, el cual arrojó como resultado HC. No se presentaron complicaciones de ningún tipo. La paciente refirió, en el control posoperatorio inmediato, la ausencia de los síntomas asociados a la ATM. Se continuó el tratamiento de ortodoncia manteniendo la dimensión vertical obtenida con la reubicación del disco articular.

A los 18 meses, se realizó el control posquirúrgico tardío y se observó una evolución satisfactoria en el examen funcional; la paciente niega dolor en apertura máxima (EVA = 0), lo que ha generado un bienestar en cuanto a su autoestima y desenvolvimiento social. Con el examen de la ATM, se evidenció una apertura bucal conservada, sin dolor, cuantificada en 46 mm, desviación de 4 mm hacia la derecha en apertura máxima. Durante la palpación y la auscultación no se hallaron ruidos ni crepitaciones. El examen intrabucal mostró aparatología ortodóntica en etapa de finalización, con línea media dental maxilar centrada, línea media dental mandibular desviada 2 mm hacia la izquierda, relación canina y molar clase I según Angle. 

**Figura 2:**
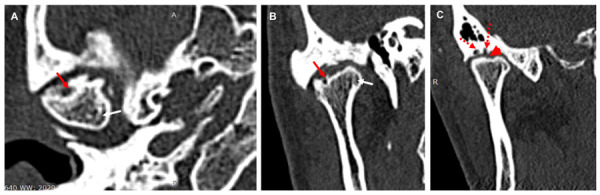
Imágenes posquirúrgicas de tomografía computarizada del caso 1. A. Corte axial de cabeza mandibular derecha mostrando densidad ósea heterogénea, imagen hipodensa bien definida, sugestiva de quiste subcondral (flecha roja), línea hiperdensa que sugiere clivaje o remodelación ósea (flecha blanca). B. Corte coronal donde se observan las mismas imágenes. C. Se observa imagen hipodensa en la cabeza mandibular sugerente de erosión (cabeza de flecha), flechas rojas punteadas indica imagen hipodensa sugestiva de quiste subcondral en fosa mandibular.

**Figura 3:**
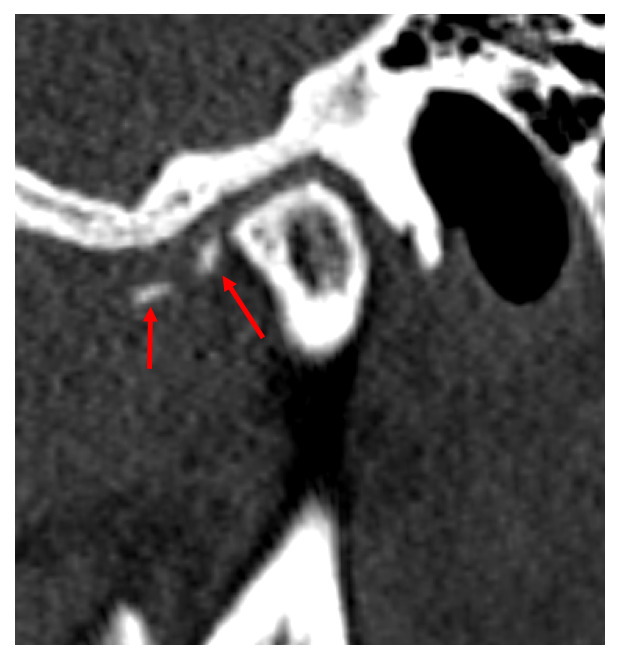
Imagen posquirúrgica de tomografía computarizada del caso 1. En plano sagital se observan imagen hiperdensa (flechas rojas) por delante de cabeza mandibular, en región correspondiente a músculo pterigoideo lateral, que sugieren fragmentos óseos.

Se realizó una TC de control y se observaron imágenes hipodensas en cabeza y fosa mandibular del lado derecho, sugestivas de erosiones (Figura 2). Hacia la vertiente posterior de tubérculo articular, fosa mandibular y borde superior de la cabeza mandibular se evidencian imágenes hipodensas, redondeadas bien definidas sugestivas de quistes subcondrales, línea hiperdensa hacia polo interno sugestiva de remodelación ósea. Por delante de la cabeza mandibular, en la región correspondiente al músculo pterigoideo lateral, se observó una imagen hiperdensa sugestiva de fragmento óseo (Figura 3). Las estructuras óseas de la ATM del lado izquierdo se encontraron dentro de los patrones imagenológicos de normalidad.

### Caso 2

Paciente femenina de 23 años, sin antecedentes médicos contributorios, quien refiere el inicio de la enfermedad actual a los 18 años, cuando evidenció un crecimiento asimétrico de la mandíbula, motivo por el cual acudió a consulta de facultativo en el año 2018, quien diagnosticó DDE clase III. Al no poder costear el tratamiento, acudió al SCM para la evaluación e instauración de la terapéutica.

En el examen funcional, la paciente presentó asimetría facial, deflexión hacia la derecha, desviación mandibular hacia la derecha, refirió dolor moderado (EVA = 7) al acto masticatorio de alimentos sólidos y en apertura máxima, se evidenciaron ruidos en apertura y cierre en ambas articulaciones, y alteración de la oclusión. Al examen de la ATM, la paciente presentaba ruidos crepitantes en apertura temprana y cierre tardío bilateral, clic en apertura tardía y cierre tardío bilateral, deflexión hacia la derecha en apertura de 1 mm, desviación hacia la izquierda en cierre. Apertura bucal conservada cuantificada en 45 mm, con dolor en apertura máxima. 

**Figura 4:**
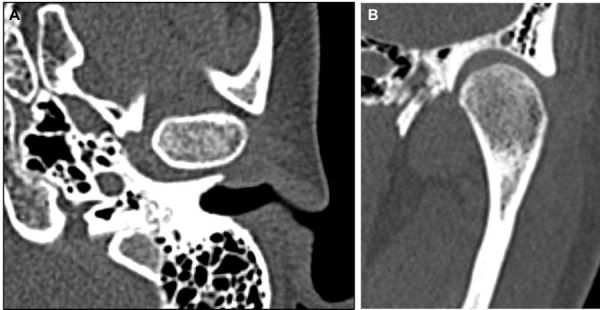
Imágenes prequirúrgicas de tomografía computarizada del caso 2. A. Corte axial de cabeza mandibular izquierda, presentado densidad ósea homogénea. B. Corte coronal donde se evidencian densidad ósea normal y aumento de volumen a nivel de cabeza mandibular.

Durante el examen clínico intrabucal se observó relación molar clase III bilateral y canina clase I bilateral, línea media dental maxilar centrada con respecto a línea media facial, línea media dental mandibular desviada 1 mm con respecto a línea media facial. El examen imagenológico con TC reveló hiperplasia condilar izquierda (Figura 4) y deformidad dentoesqueletal clase III asimétrica. En la ATM del lado derecho no se observaron cambios en la densidad ósea ni en las características morfológicas.

Se planificó cirugía ortognática en la que se realizó osteotomía Le Fort I de impactación posterior para nivelación, CA izquierda de 8 mm, osteotomía sagital de rama bilateral y mentoplastia de corte oblicuo de impactación izquierda de 5mm y avance de 3 mm. El estudio histopatológico del cóndilo mandibular concluyó la presencia de HC. 

No se presentaron complicaciones de ningún tipo. La paciente no refirió síntomas asociados con la ATM afectada posterior a la cirugía. Se continuó el tratamiento de ortodoncia para preservar una oclusión estable y evitar recidiva.

A la fecha de este estudio, la paciente cuenta con tiempo posoperatorio de 28 meses. Al examen funcional se observó su evolución satisfactoria, sin dolor en apertura bucal máxima (EVA = 0). Durante el examen clínico de la ATM, se evidenció una apertura bucal de 45 mm sin dolor, desviación en apertura tardía de 3 mm hacia la izquierda. En examen clínico intrabucal se observó aparatología ortodóntica en oclusión estable con línea media dental maxilar desviada 3 mm hacia la izquierda, línea media dental mandibular centrada con respecto a línea media facial, ambas con respecto a línea media facial, relación canina y molar clase I según Angle. 

**Figura 5:**
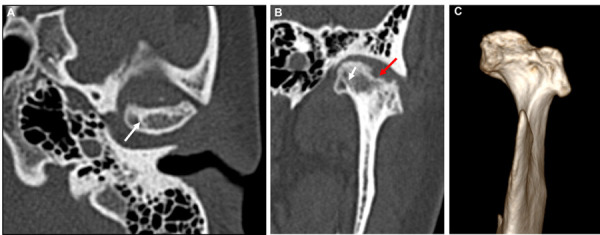
Imágenes posquirúrgicas de tomografía computarizada del caso 2. A. Corte axial de cabeza mandibular izquierda mostrando línea hiperdensa sugestiva de remodelación ósea (flecha blanca). B. Corte coronal donde se evidencia la línea hiperdensa que sugiere remodelación ósea (flecha blanca), se observa zona de erosión hacia borde superior (flecha roja). C. Imagen coronal reconstruida volumétricamente en 3D mostrando cambios en las características morfológicas.

**Figura 6:**
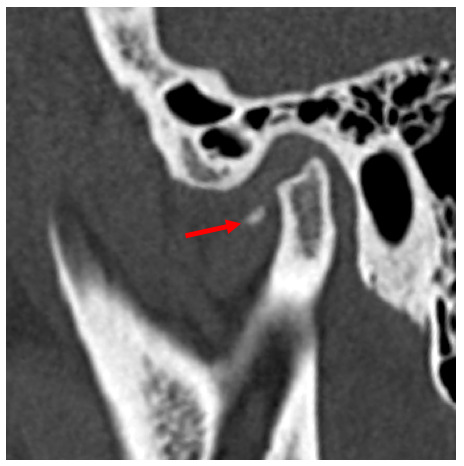
Imagen posquirúrgica de tomografía computarizada del caso 2. En plano sagital, se observan imagen hiperdensa (flecha roja) por delante de cabeza mandibular, en región correspondiente a músculo pterigoideo lateral, sugestiva de fragmento óseo.

Se complementó el control posquirúrgico tardío por medio de TC. Se observó imagen hipodensa sugestiva de erosión hacia borde superior de cabeza mandibular izquierda y línea hiperdensa hacia polo interno sugestiva de remodelación ósea (Figura 5). Por delante de la apófisis condilar, en la región correspondiente al músculo pterigoideo lateral del lado izquierdo, se evidenció imagen hiperdensa sugestiva de fragmento óseo (Figura 6). 

## DISCUSIÓN

La HCU es una patología no neoplásica y autolimitante caracterizada por el crecimiento excesivo del cuello y la cabeza condilar ^(1, 2)^ que causa asimetría facial, ocurre entre los 10 y 30 años, con mayor frecuencia en el sexo femenino ^1^^,^^4^^-^^6^. Aunque la etiología es desconocida, se ha relacionado con trauma, infección articular, desórdenes hormonales o endocrinos, hipervascularización del cóndilo, cambios intrauterinos y factores genéticos ^2^^,^^5^^,^^7^^,^^19^.

El cartílago condilar es considerado el centro del crecimiento craniofacial y presenta cuatro capas distintivas, a saber: un revestimiento de tejido conectivo (capa de fibrocartílago), una capa de tejido mesenquimal indiferenciado (proliferativa), una capa transicional y una capa de cartílago hipertrófico, las mismas que tienen un espesor que varía entre 0,18 y 0,48 mm ^20^. La actividad de la capa proliferativa parece regular la tasa a la cual la cabeza y el cuello condilar crecerán; en cóndilos normales, la formación de cartílago y el reemplazo del cartílago por hueso cesa alrededor de los 20 años, de manera que la cavidad medular está enteramente ocluida desde el cartílago remanente por la placa de hueso. La incapacidad de cierre de esta placa en presencia de una capa proliferativa activa se cree que es el mayor factor etiológico de una HC ^8^^,^^21^. 

La HCU puede influenciar el tamaño y la morfología de la mandíbula, afectar la oclusión e, indirectamente, el maxilar, con el desarrollo o empeoramiento de las DDF, asimetría facial y dolor en la ATM ^1^^,^^22^. De manera que esta patología altera no solo la estética facial del paciente, sino también su función masticatoria ^5^^,^^8^. Los casos incluidos en este reporte se observaron en pacientes del sexo femenino en la tercera década de la vida. Clínicamente, ambas mostraron signos/síntomas de disfunción temporomandibular, desviación de la mandibula durante la apertura y cierre, laterodesviación de la sínfisis mandibular hacia el lado no afectado y el patron esqueletal clase III. Asimismo, se evidenció el agrandamiento tridimensional de un lado de la mandíbula que incluía el aumento de tamaño de la cabeza y cuello condilar y la rama ascendente, la anomalía terminaba en la sínfisis del lado afectado, características que son reportadas en la literatura ^1^^,^^5^^,^^8^^,^^10^^,^^13^. 

La desición sobre la terapéutica a instaurar depende de la edad del paciente, el grado de asimetría, el tipo de maloclusión y, principalmente, de la actividad del crecimiento condilar ^1^^,^^2^^,^^5^^,^^12^^,^^13^. La CA es el procedimiento de elección en casos con HCU activa, en este se elimina alrededor de 5-8 mm de la superficie superficie superior de la cabeza del cóndilo, incluidos los polos medial y lateral, con lo que se detiene el crecimiento excesivo al retirar el centro de crecimiento condilar. Si esta terapéutica se aplaza, el paciente podría sufrir disfunción masticatoria y del habla, lo que empeoraría la configuración estética ^23^. La CA ha sido combinada con cirugía ortognática; en este sentido, se ha señalado que se obtiene un resultado predecible y estable cuando ambas terapéuticas son utilizadas, en comparación con la cirugía ortognática empleada como tratamiento único ^1^^,^^2^^,^^5^^,^^12^^,^^13^^,^^23^.

Debido a la edad de las pacientes y a la imposibilidad de un examen más exaustivo mediante medicina nuclear, se decidió realizar una CA acompañada de cirugía ortognática con la finalidad de corregir la asimetría facial, la maloclusión y el posicionamiento del disco articular. La presencia de una clase III esqueletal orientó la combinación de los procedimientos quirúrgicos para obtener resultados funcionales y estéticos. En el seguimiento posoperatorio tardío, ambas pacientes presentaron apertura bucal preservada, no manifestaron dolor o ruido articular, se constató un leve desvío mandibular hacia el lado intervenido, lo que es esperado en este tipo de cirugías y se verificó una oclusión en relación molar y canina clase I según Angle. 

Se han observado cambios posquirúrgicos en los cóndilos intervenidos por HC. En este sentido, estudios experimentales en modelos animales han indicado que las cargas en el disco articular y la edad del sujeto son factores importantes en la regeneración condilar ^24^^-^^28^. La presencia de un disco intacto parece influenciar dicha regeneración, en vista de que no se ha observado remodelación en cóndilos tratados con condilectomía y distectomía ^24^; asimismo, el potencial de regeneración es mayor en individuos jóvenes que en adultos ^29^. 

Mediante el estudio con TC, se observó en el cóndilo intervenido de ambas pacientes estudiadas la presencia de líneas hiperdensas en el muñón de la cabeza mandibular, sugestivas de remodelación ósea, localizadas hacia el polo medial, a diferencia de Katsumata *et al*. ^14^, quienes las evidenciaron en la parte posterior, medial y media; los autores definieron estas líneas como remodelación ósea adaptativa. Este doble contorno fue observado por Suei *et al*. ^30^. Histológicamente, constituye una nueva capa de hueso fuera del margen óseo original, que se origina de la formación perióstica de hueso o de la osificación endocondral en la parte posterior de la cabeza mandibular. Probablemente, esta diferencia en la localización de las líneas se deba a los distintos tratamientos previos realizados. Las primeras son consecuencia de una cirugía que invade el espacio articular y las observadas por los autores referidos se deben a cambios en la posición condilar mediante dispositivos intrabucales que ocasionaron tensiones en el espacio articular. 

Asimismo, fueron observadas imágenes erosivas y quistes subcondrales, tanto en la cabeza mandibular como en la fosa mandibular, las cuales imagenológicamente son sugestivas de procesos degenerativos; estos hallazgos fueron observadas por Rojare *et al*. ^9^. Los cambios pueden ser consecuencia del corte quirúrgico que, al dejar el hueso medular expuesto hacia el espacio articular, propició la retención del líquido sinovial entre los espacios trabeculares superficiales ocasionando la imagen sugestiva de quiste subcondral. 

También fue evidenciado un tipo de crecimiento de apariencia exofítica, localizado por encima de los polos de la cabeza mandibular. Esta misma observación fue relatada por Rojare *et al*. ^9^, quienes lo definieron como un reordenamiento de tipo exofítico, lo cual coincide con lo afirmado por Gallagher *et al*. ^31^, los cuales describieron el hallazgo como una remodelación dismórfica posquirúrgica.

El tiempo de este crecimiento condilar coincide con lo afirmado por Fariña *et al*. ^15^ y Gallagher *et al*. ^31^, quienes indicaron que después de la condilectomía fue observada una remodelación condilar a los 12 meses del procedimiento para igualar gradualmente al lado contralateral sano. Los exámenes imagenológicos de ambos casos fueron realizados a los 18 meses y 28 meses, respectivamente. En ninguna de las pacientes se observó signos imagenológicos de esclerosis, a diferencia de lo reportado por Rojare *et al*. ^9^, quienes evidenciaron este tipo de imagen previo a la cirugía y en el posquirúrgico, tanto en el lado sano como en el patológico.

Por otra parte, Rojare *et al*. ^9^ observaron calcificaciones localizadas por delante de la cabeza mandibular intervenida, sugestivos de dos fragmentos de hueso. Este hallazgo fue observado en las pacientes de nuestro estudio, probablemente producto de pequeñas esquirlas resultantes del corte óseo. En ambas pacientes, los cóndilos contralaterales al cóndilo hiperplásico mostraron un patrón imagenológico dentro de la normalidad, a diferencia de lo referido por Rojare *et al*. ^9^, quienes reportaron la presencia de quistes subcondrales y erosiones en el cóndilo contralateral, cuyas imágenes desaparecieron en los controles imagenológicos posquirúrgicos. 

## CONCLUSIONES

La HCU puede ser tratada eficazmente con CA acompañada de cirugía ortognática, con lo que se obtienen resultados estéticos y funcionales. Las pacientes tratadas refirieron estar asíntomáticas, con una apertura bucal en el rango de la normalidad y una relación molar y canina clase I según Angle en el posoperatorio tardío. Al examen imagenológico mediante tomografía computarizada se evidenció remodelado óseo en los cóndilos intervenidos, lo que parece indicar que la cabeza mandibular mantiene su capacidad adaptativa aún en pacientes adultos; los cóndilos contralaterales se encontraban dentro del patrón imagenológico de normalidad. 

## Fuente de financiamiento:

Autofinanciado
